# Individual‐based analyses reveal effects of behavioral and demographic variables associated with multi‐annual reproductive success of male and female lake sturgeon

**DOI:** 10.1002/ece3.10253

**Published:** 2023-07-12

**Authors:** Thuy‐Yen Duong, James Bence, Patrick S. Forsythe, James A. Crossman, Edward A. Baker, Nicholas M. Sard, Kim T. Scribner

**Affiliations:** ^1^ Department of Fisheries and Wildlife Michigan State University East Lansing Michigan USA; ^2^ Department of Zoology Michigan State University East Lansing Michigan USA; ^3^ Michigan Department of Natural Resources Marquette Michigan USA; ^4^ Biological Sciences Department SUNY Oswego Oswego New York USA; ^5^ Present address: College of Aquaculture and Fisheries Can Tho University Can Tho City Vietnam; ^6^ Present address: Department of Natural and Applied Sciences University of Wisconsin – Green Bay Green Bay Wisconsin USA; ^7^ Present address: Fish and Aquatics, BC Hydro Castlegar British Columbia Canada

**Keywords:** individual‐based analysis, lake sturgeon, mating behavior, parentage analysis, reproductive success

## Abstract

Quantifying effects of individual attributes and population demographic characteristics that affect inter‐ and intrasexual interactions and adult reproductive success, and the spatial and temporal contexts in which they are expressed is important to effective species management. Multi‐year individual‐based analyses using genetically determined parentage allowed the examination of variables associated with the reproductive success of male and female lake sturgeon (*Acipenser fulvescens*) in the well‐studied population in Black Lake, Michigan, USA. Spawning lake sturgeon (a total of 599 individuals where many were captured more than once based on 1024 total captures) and larvae (*N* = 3436) were genotyped during each of seven consecutive years (2001–2007). Factors associated with individual reproductive success differed between sexes and varied among spawning groups within a year and among years depending on spawning date (higher reproductive success earlier in the season for females) and spawning locations (higher reproductive success in upstream spawning zones for females). Female reproductive success increased nonlinearly with increasing body size. Male reproductive success increased with increasing residence time in spawning areas and, to a modest degree, with increasing body size in a nonlinear fashion. Fixed effects of repeatability in spawn timing and location across years led to consistently higher or lower reproductive success for females. Results identified factors, including time spent at spawning areas by males and intersexual encounters and mate number, that contributed to higher interindividual variance in reproductive success and affected population levels of recruitment, the degree of subpopulation genetic structure (lack of isolation by time), and effective population size.

## Graphical abstract

We use multi‐locus genotypes and genetic determination of parentage for 599 adult and 3436 offspring lake sturgeon over 7 years to estimate male and female reproductive success (RS) and apportion variation RS into biotic and environmental factors using a novel mixed model analysis. Fixed effects of repeatability in spawn timing and location across years led to consistently higher or low RS at the individual level for males and females. Results identify the importance of factors, including male mating behavior associated with intersexual encounters and mate number, that contributed to interindividual variance in RS and population levels of recruitment, the degree of subpopulation genetic structure (lack of isolation by time), and effective population size.

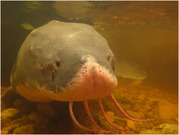

## 1 INTRODUCTION

Polygamous mating occurs in many taxa including fish (Briton et al., 1994; Hernaman & Munday, 2007) but is difficult to observe directly. In complex mating systems of polygamous fish, interindividual variation in reproductive success (defined here as the number of offspring attributed to a parent that survives to the period of larval dispersal from the spawning areas 5–35 days posthatch; Duong, Scribner, Crossman, Forsythe, Baker, Kanefsky, et al., 2011; Duong, Scribner, Crossman, Forsythe, Baker, & Magnan, 2011) has been attributed to many factors (Avise et al., 2002; Kokita & Nakazono, 1998). For example, within a population, reproductive success can vary as a function of the distribution and abundance of potential mates (Emlen & Oring, 1977) and the availability and location of essential resources (e.g., food and spawning locations; Hernaman & Munday, 2007; Verner & Willson, 1966). The distribution and abundance of each sex can vary over time and space (Shuster & Wade, 2003) due to differences in the timing of arrival to breeding sites between males and females (Kokita & Nakazono, 1998; Seamons et al., 2004) or due to differences in interannual breeding periodicity (Forsythe et al., 2012; Larson et al., 2020). Accordingly, temporal and spatial variation in adult sex ratio and abundance can influence the number of mates (e.g., for females, Allee effects associated with density‐dependent mating success and probabilities of gamete fertilization). Paternal factors also contribute to the probabilities of egg fertilization (Kamler, 2005; Trippel & Neilson, 1992). For example, positive relationships between sperm density and/or motility and egg fertilization rates were reported in several fish species such as bluehead wrasse (*Thalassoma bifasciatum*; Petersen et al., 2001) and bluegill sunfish (*Lepomis macrochirus*; Neff et al., 2003). While behavioral tactics clearly impact reproductive success, the impacts of behavioral tactics employed by males and females with polygamous mating systems on population levels of recruitment and genetic diversity are largely unknown.

Following fertilization, many factors affect reproductive success, that vary widely over taxonomically diverse fishes. Parental effects and behavior and environmental conditions associated with the location and timing of reproduction play important roles in offspring viability and population levels of recruitment (Kamler, 2005). Maternal phenotypic traits such as body size and age have also been documented to affect offspring body size, growth, and survival (Chambers & Leggett, 1996; Heins et al., 2004; Kamler, 2005). For example, female body size is positively related to egg and larval size and negatively related to the probability of larval mortality due to starvation and predation (Kamler, 2005). Female behavior including the selection of spawning date and location will determine the environmental conditions experienced by eggs and larvae (Einum & Fleming, 2000; Hendry & Day, 2005; Jørgensen et al., 2008), and therefore can affect reproductive success, through effects on offspring survival during early life stages.

In broadcast spawning species, reproductive success likely varies as a function of spawner density (Moller & Legendre, 2001; Rowe & Hutchings, 2003). When a population is at low abundance, male reproductive success will likely decrease due to reduced mating opportunities (Levitan, 2004; Moller & Legendre, 2001; Rowe & Hutchings, 2003). Female reproductive success is also expected to decrease due to sperm limitation (Levitan, 2004; Levitan & Petersen, 1995; Marshall & Evans, 2005). Therefore, spawning synchrony and mate availability can be important for broadcast spawning species to increase fertilization rates and ultimately reproductive success (Coma & Lasker, 1997; Emlen & Oring, 1977; Levitan & Petersen, 1995). Accordingly, broadcast spawning species may exhibit a variety of spawning behaviors (e.g., modifying arrival time at spawning sites), as observed in nest spawning species (e.g., steelhead trout; *Oncorhynchus mykiss*; Seamons et al., 2004) to acquire high‐quality spawning locations. Resource expenditures by males, for example, based on the duration of occupancy of spawning areas and the number of intersexual interactions, have also been tied to male reproductive success in lake sturgeon (*Acipenser fulvescens*; Larson et al., 2020).

Lake sturgeon (Figure S1) is a broadcast spawning species characterized by extreme longevity (>100 years) and iteroparity (Auer, 1999). Observational studies describing spawning behavior indicated that lake sturgeon spawn in groups where one female can be surrounded by several males, and each male has the potential to mate with multiple females (Bruch et al., 2016; Bruch & Binkowski, 2002). Spawning occurs when ovulating females arrive at spawning sites. During a spawning bout, eggs and sperm are released simultaneously into the water column. Eggs are typically deposited by an individual female episodically with multiple males lasting 8–12 h (Bruch & Binkowski, 2002) and can extend several days if environmental conditions are unsuitable (Dammerman et al., 2019).

Lake sturgeon spawning runs are usually male‐biased due to differences in interspawning interval (females longer than males; Forsythe et al., 2012). Sex ratios can vary by year and among spawning locations within a population (Auer, 1999). In addition, the size and composition of spawning aggregations can vary within a spawning season, as different individuals enter and leave spawning areas in response to different water temperatures and discharge regimes (Dammerman et al., 2019; Forsythe et al., 2012), mate availability (Larson et al., 2020), and the arrival of additional potential mates. Lake sturgeon do not provide postovulatory parental care (Bruch & Binkowski, 2002), contributing in part to high mortality in early life stages (e.g., survival rates from eggs to age 0 juvenile stage <0.1%; Caroffino et al., 2010; Crossman et al., 2018; Forsythe et al., 2018). High and variable mortality during early life stages may result in high variation in reproductive success among individuals depending in part on when and where an individual spawns and features of the river environment (e.g., river flow, temperature, and spawner abundance). Differences in adult abundance and sex ratios characterizing early and late spawning groups may be consistent across years, potentially resulting in predictable interindividual variation in reproductive success across years. Alternatively, individual reproductive success may be inconsistent across years because consistent spawning behavior does not confer a consistent outcome. Thus, the spawning behaviors of lake sturgeon make this species an interesting subject to examine hypotheses of how behavioral, biotic, and abiotic factors affect male and female reproductive success within and among years.

Our objective was to quantify male and female lake sturgeon reproductive success and to evaluate whether reproductive success varied as a function of variables tied to individual characteristics including body size, timing of spawning, spawning location, and reproductive behaviors (e.g., intermale variation in length of spawning site occupancy that affect intrasexual and intersexual interactions). Using parentage analysis based on microsatellite genotypes of adults and offspring over a 7‐year period (2001–2007), we quantified the number of offspring produced (reproductive success) for each adult during one or more years that individuals were captured while spawning. We developed novel mixed effects analyses to account for uneven sampling among years and different interbreeding intervals among individuals. Multi‐year individual‐based analyses, in which the same individuals were captured and recaptured across several years, allowed us to quantify the degree of inter‐ and intraannual variability in the effects of factors contributing to male and female reproductive success. Given the high degree of individual repeatability in male and female spawning date and location (Forsythe et al., 2012), consistency in the magnitude of interindividual variation in reproductive success would provide evidence for reproductive isolation among early and late spawning groups (Hendry & Day, 2005; Tomaiuolo et al., 2007) and could account for interannual variation in effective breeding numbers (*N*_b_) and generational estimates of effective population size (*N*_e_, Duong et al., 2013).

## 2 MATERIALS AND METHODS

### 2.1 Study site

**FIGURE 1:**
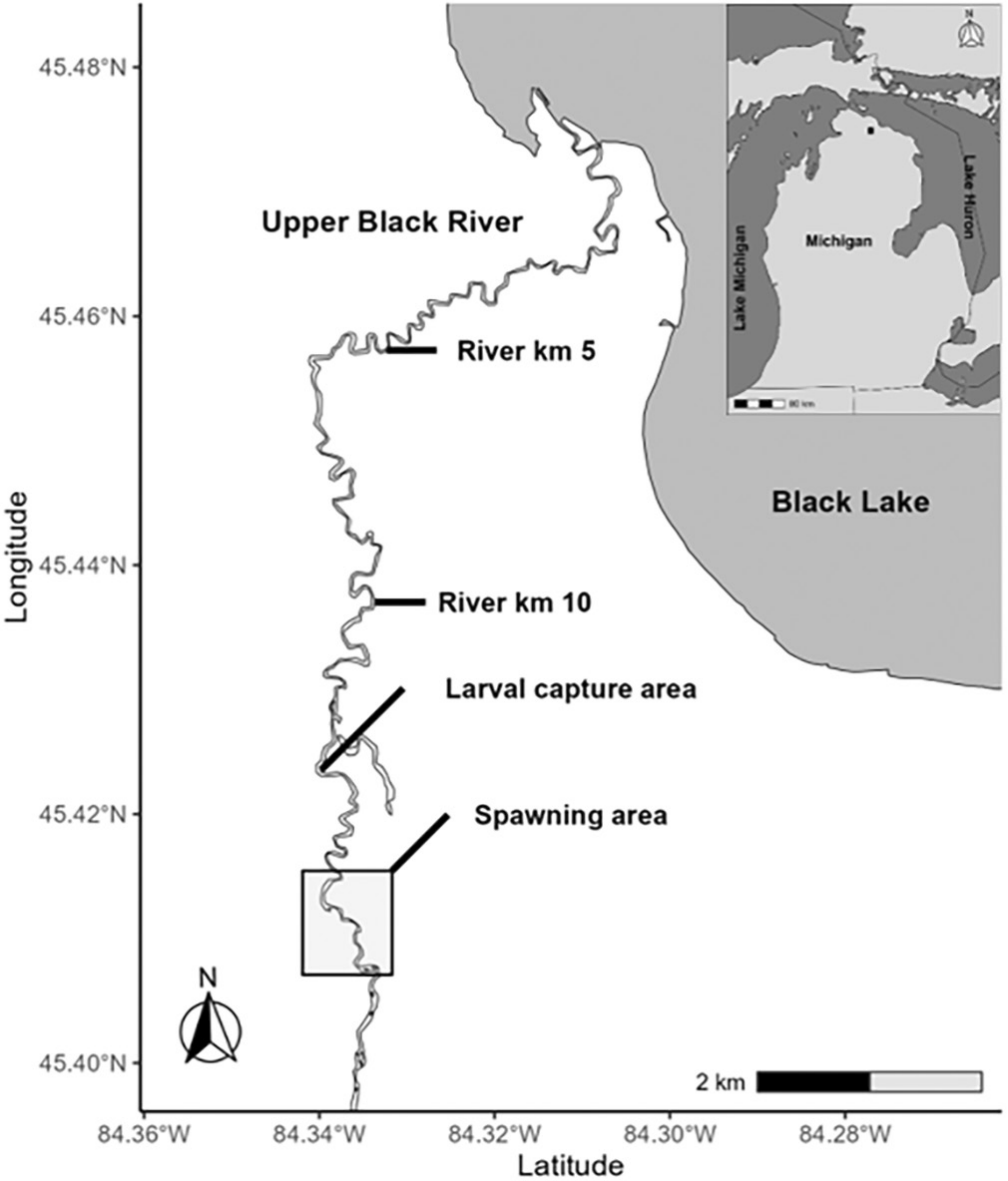
Study site on the Upper Black River, Michigan (MI), USA, showing positions of adult spawning areas and larval collection sites and an enlarged view of the six spawning zones.

Our study was conducted in the Upper Black River (UBR), the largest tributary to Black Lake, Michigan, USA (latitude 45°43′N, longitude 84°15′W; Duong, Scribner, Crossman, Forsythe, Baker, & Magnan, 2011; see Figure 1), and the only tributary used for spawning by lake sturgeon in this drainage. The lake sturgeon population in Black Lake is isolated from other populations in adjacent lakes by dams (Smith & Baker, 2005). Adults spawn over a 1.5 km section of the UBR. Previously, we designated six zones of spawning activity within this section, hereafter referred to as “reproductive zones,” which were used consistently across the study period (Figure 1; Duong, Scribner, Crossman, Forsythe, Baker, & Magnan, 2011; Forsythe et al., 2012). The relatively small size of the river (generally ~25 m in width) and shallow spawning areas (most ~1 m in depth) allowed most adults to be observed and captured to collect phenotypic data and tissue samples for genetic determination of parentage.

### 2.2 Sample collection

#### 2.2.1 Adult field collection

Adults and larvae were sampled and genotyped during seven consecutive years (2001–2007; Table 1, see also Duong, Scribner, Crossman, Forsythe, Baker, Kanefsky, et al., 2011; Duong et al., 2013). Sampling for adults was conducted by wading the length of stream (~1.5 km; Figure 1) encompassing all reproductive zones one or more times per day during the entire spawning season (typically late April to early June) each year. Spawning adults were captured using long‐handled dip nets. Sex was determined by the extrusion of gametes. Adult males and females were tagged using 134.2 kHz passive integrated transponder (PIT) tags and externally using Floy tags. Floy tag colors were unique to a year and to the spawning period (early vs. late in the time of spawning) and were unique for males and females so field personnel could identify sex (males tagged on the right dorsal surface adjacent to the dorsal fin and females tagged on the left side).

**TABLE 1:** Summary of the number and proportion of the total spawning adult lake sturgeon population captured and sex ratio yearly.

Year	Captured adults				Total no. captured	Total % captured	Adult sex ratio (Male:Female)	No. larvae collected	No. larvae genotyped	% Larvae genotyped
	No. males	% Males	No. females	% Females						
2001	71	0.561	44	0.799	115	0.633	1.61	1691	553	32.7
2002	71	0.49	33	0.546	104	0.506	2.15	1320	136	10.3
2003	80	0.492	40	0.607	120	0.525	2.00	16,417	358	2.2
2004	76	0.423	25	0.352	101	0.403	3.04	437	241	55.1
2005	106	0.541	47	0.619	153	0.563	2.26	7800	362	4.6
2006	162	0.765	63	0.78	225	0.769	2.57	5587	342	6.1
2007	143	0.629	63	0.736	206	0.658	2.27	1444	144	100
Total	709	0.568	315	0.637	1024	0.587	Mean 2.27	34,696	3436	Mean 9.9

*Note*: The number of larval lake sturgeon captured and the proportion of captured larval lake sturgeon genotyped is also provided.

Biological data were collected from each adult captured each year. A dorsal fin clip (~1 cm^2^) was collected for genetic analysis. All individuals were measured for weight (kg), fork length (from snout to fork of caudal fin; FL), and total length (from snout to tip of caudal fin; TL; cm). Temporal variation in spawning date is widely observed in lake sturgeon (e.g., Kessel et al., 2018). We also recorded the date and reproductive zone of each capture (Figure 1). The date defining the end of the early period was year‐specific and based on intervals between groups of newly arriving adults (for details see Forsythe et al., 2012). Because females spent only a few hours or days at the spawning habitat (hereafter referred to as spawning grounds; Dammerman et al., 2019; Forsythe et al., 2012), the date and location of capture were assumed to be the date and location of spawning. Male spawning behavior varied in response to the number and location of spawning females (Dammerman et al., 2019; Forsythe et al., 2012; Larson et al., 2020). Thus, for a given offspring captured, we determined the identity of the male and female parents by genetic parentage analysis. The location where the male spawned with this female was specified as the location (reproductive zone; Figure 1), where the female was captured. Males with reproductive success of zero were not included in the analysis of explanatory factors because the timing of any reproductive behavior could not be ascertained.

We estimated the abundance of spawning adults for each year of the study from the total abundance of males and females in the population and spawning return‐time probabilities (Pledger et al., 2013). The Pledger et al. (2013) model is a modified Cormack‐Jolly‐Seber model that uses spawning run mark and recapture data to estimate total population abundance for males and females separately and produces estimates of spawning return‐time probabilities. Based on Black River spawning run data from 2001 to 2022 spawning return‐time probabilities for males are 0.41 and 0.59 for spawning in years *K* + 1 and *K* + 2 given spawning in year *K*. For females spawning return‐time probabilities are 0, 0.06, 0.57, 0.25, and 0.11 for spawning in years *K* + 1 to *K* + 5 given spawning in year *K*. To estimate the proportion of the males and females spawning in any given year during the study, we initially set a starting abundance of 1000 males and 1000 females and used the return‐time probabilities to calculate spawner abundance for 25 consecutive years. For males, spawner abundance in year 1 of the simulation was set at 500 (half of the total abundance), and for females, spawner abundance was set at 250 for years 1 and 2 of the simulation. Following some initial variability in annual abundance estimates, the abundance of males spawning each year stabilized at 629 or 62.9% of all males. At year 25 of the simulation the estimated abundance of females spawning each year was 274 or 27.4% of all females. These proportions were then used to estimate total spawner abundance for 2001–2007 by multiplying with the total population abundance of males and females from the Pledger et al. (2013) model estimates.

#### 2.2.2 Larval field collection

Larval sampling was conducted at night each year starting approximately 10 days after the first spawning event and continuing for 25–40 days until no larvae were captured for two consecutive nights. We define the larval stage as starting at the time of absorption of the yolk sac and initiation of exogenous feeding, through the period of passive dispersal (drift) from upstream spawning areas to the time of settlement in downstream areas of the river. The larval sampling protocol (Smith & King, 2005) was consistent across years and involved the deployment of five D‐frame drift nets spread at equidistant intervals across the river (~25 m width) nightly throughout the larval drift period. Nets were placed approximately 1.5 km downstream from the furthest downstream spawning area. Therefore nets collected larvae from all reproductive zones. The spawning zone and timing of spawning of adults of each offspring were established based on adult parentage assignment. Nets were checked hourly from 21:00 to 02:00 (details in Receveur et al., 2022). Based on total river discharge measured nightly, and total discharge passing through the five D‐frame drift nets, we estimated that nets sampled an average of ~13% of all dispersing larvae nightly. The total body length of larvae at the time of capture was estimated to be (mean ± SD) 20.1 ± 1.58 mm (min–max 15.1–24.6 mm; J. Riedy, unpublished data). Captured larvae were transferred to a streamside hatchery where they were reared for several months (late August). Before releasing the fish to the wild at approximately 15 cm total length, a small portion of the caudal fin was clipped from each fish for genetic analysis. Mortalities during the rearing period were preserved in 95% ethanol. Within a year, larval samples used for microsatellite genotyping (Table 1) were stratified by sampling night and randomly selected nightly from the number of preserved fin clips and hatchery mortalities each year. The larval drift numbers and number subsampled each year for genetic analysis varied substantially due to annual natural conditions and available budget and staff time, respectively (see details in Duong et al., 2013).

### 2.3 Genetic analysis

We genotyped samples from all captured adults and selected larval samples at 12 tetra‐nucleotide microsatellite loci including *Spl 120* (McQuown et al., 2000); *AfuG 68B* (McQuown et al., 2002); *Aox 27* (King et al., 2001); *AfuG 68*, *AfuG 9*; and *AfuG 63*, *AfuG 74*, *AfuG 112*, *AfuG 56*, *AfuG 160*, *AfuG 195*, and *AfuG 204* (Welsh et al., 2003). Genomic DNA was extracted from adult and larval samples using the QIAGEN DNeasy® Blood and Tissue Kits (QIAGEN, Inc.). Conditions for locus amplification by polymerase chain reaction (PCR) and genotype quality assurance (independent scoring by two trained lab staff and 10% regenotyping error check) were as described in Duong, Scribner, Crossman, Forsythe, Baker, Kanefsky, et al. (2011) and Duong et al. (2013).

### 2.4 Parentage analysis

Use of multiple parentage analysis programs that are based on different statistical properties was recommended to increase parentage assignment accuracy (Jones et al., 2010; Lee, 2008; Walling et al., 2010). Previous work by our group has embraced this philosophy for the years of this study (e.g., Duong et al., 2013). We conducted parent pair assignment to larvae using two categorical allocation programs including the Parentage Allocation of Singles on Open Systems (PASOS) program, version 1.0 (Duchesne et al., 2005) and a likelihood‐based software, CERVUS version 3.0 (Kalinowski et al., 2007). Details of user‐defined parameters for each program and criteria used for selecting putative parent‐offspring allocations for Black River lake sturgeon are described in Duong, Scribner, Crossman, Forsythe, Baker, Kanefsky, et al. (2011); Duong, Scribner, Crossman, Forsythe, Baker, and Magnan (2011); Duong et al. (2013). Power statistics calculated as part of a CERVUS analysis were used to evaluate the accuracy of parent‐offspring assessments. The mean number of alleles observed and expected heterozygosity were also calculated. Parent pair allocations for individual larvae that were concordantly assigned from the two programs were used in the calculation of male and female reproductive success. Reproductive success for a male and female individual each year was defined as the number of larval offspring assigned to that individual.

### 2.5 Statistical analysis

We developed statistical models that related reproductive success to explanatory factors and accounted for annual asymmetry in samples processed, interadult variation in the number of years spawned, and overdispersion of data. Our strategy for model selection and parameter estimation first involved the choice of a random effects model for each sex, before selecting the best fixed effect models. Our primary analysis of reproductive success was done separately for males and females because of fundamental differences in the reproductive behavior (i.e., spawning date, residence time on the spawning ground [duration of time in days between the earliest and latest spawning date of females a male has shared parentage with], spawning interval across years, the number of mates, etc.) between males and females (Oliveira et al., 2008), which argued a priori for different explanatory variables for the different sexes. Specific random effects and rationale are specified in Section 2.5.1. Specific fixed effect variables, rationale for them, and how they were calculated are given in Section 2.5.2.

#### 2.5.1 The random portion of statistical models for reproductive success

Our basic modeling approach for the evaluation of reproductive success treated the number of offspring produced by an individual as being Poisson distributed, potentially with overdispersion. Modeling count data with a Poisson distribution is perhaps the most common approach in ecology (Bolker et al., 2009) but often such data are overdispersed (meaning the variance is greater than rather than equal to the mean as assumed by the Poisson distribution; Hilbe, 2011). We allowed for overdispersion by considering models that included a random effect for individual observations (Obs), a simple approach to potential overdispersion that has been shown to have robust performance (Harrison, 2014). Because of the long‐term nature of this study, the same individual adults were observed for multiple years. We therefore also considered a random effect of the individual (Ind).

Although sampling protocols were consistent across years, and lake sturgeon have high individual repeatability in reproductive location and timing (Forsythe et al., 2012), catchability still might have varied over years, and sampling fraction (proportion of larvae sampled that were genotyped) also varied from year to year (Table 1; see also in Duong et al., 2013). In addition, actual reproductive success might have varied from year to year due to environmental influences that were not modeled but were known to influence survival during the egg stage (Finley et al., 2019). Consequently, we considered models that included a random year effect. Year is essentially a nuisance factor, given that it partly reflects varying catchability and the varying sampling fraction, not just variation in actual reproductive success. Flow and temperature regimes also vary annually and can influence the reproductive zone used and the timing of spawning (Forsythe et al., 2012). We chose the best random effects model as the one with the lowest AIC (when all fixed effects were in the model) to use in our primary analysis of fixed effects (Section 2.5.2). We also refitted the entire suite of fixed effect models to each random effects model that was within three AIC of the lowest when fitting to the fully saturated fixed effect model. We did not present the detailed results of these alternative analyses in the main text, but instead briefly summarized them with respect to how they informed the robustness of the primary analysis.

#### 2.5.2 Statistical models for fixed effects associated with reproductive success

Following the selection of a random effects model, we then fit each alternative model of fixed effects for the analysis under consideration, using the same previously selected random effects model in each case. Because our models potentially included a random year effect, our analyses of fixed effects were directed at how explanatory factors influenced reproductive success within a year.

To place explanatory factors on the same scale and improve model estimation ability the continuous variables were rescaled so they ranged from a minimum value of 0 to a maximum value of 1 within each year (i.e., standardized *X* = (*X*−min)/(max–min)), where *X* is the unscaled variate and min and max are the minimum and maximum of the *X* within a year. In models that included a given continuous standardized variable, both the original standardized value and its square were included as variables, thus allowing for a type of nonlinear relationship. We adopted this approach because preliminary analyses demonstrated that the full model that included quadratic terms substantially outperformed the otherwise fully saturated fixed effect model that did not (we also checked that the best [lowest AIC] model remained better than the same model with the quadratic terms dropped).

We used a log‐link function, which treated the mean reproductive success as being an exponential function of a linear combination of the predictors. For each set of models with alternative fixed effects, we presented results for the lowest AIC fixed effects model but also briefly discussed variables included and predictions from alternative fixed effect models that produced results with an AIC within three of the best‐fixed effect model (Burnham & Anderson, 2002).

For both males and females, we used continuous variables standardized by year, both because as noted above differences in the response variables among years were not biologically meaningful (as the fraction of larvae captured was not constant) and because we assumed that relative values within a year would be most predictive of differences within a year. For females, we considered the following potential fixed effect explanatory variables: standardized body size (minimum TL of females observed that year)/(maximum TL of females observed that year − minimum TL of females observed that year), where TL is unstandardized total length as a continuous factor, standardized days (the number of days past the first spawning day of the year divided by the total number of days in which spawning occurred in that year) after the start of the spawning season a female reproduced (Date) as a continuous factor, and reproductive zone (Zone, six zones as described previously; Figure 1) as a categorical factor. In the main analysis, we thus considered all possible models with and without standardized body size, date, and zone, with all models including an intercept. This led to a total of eight fixed effect models that we directly compared by AIC, including the model with only an intercept.

In the main analysis for males, we used standardized time on the spawning ground (residence time) instead of spawning date, and standardized body size was calculated as for females but using male lengths observed that year. The residence time was based on the difference between the last and first confirmed spawning date based on parentage analysis and the dates of spawning for females a male shared offspring with. In cases of a male having one mate, the residence time was the day difference between the capture date of the male and his mate. Only males with at least one offspring were included in the analysis. The residence time was used instead of a spawning date because males generally contributed offspring based on spawning with multiple females potentially over the entire spawning period rather than on a single date (Larson et al., 2020). Although there is a known relationship between residence time and male body size (Larson et al., 2020), we used both standardized body size and (standardized) residence time because these variables were only weakly correlated (*r* = .07, *p* = .12), the correlation was still weak but stronger for unstandardized values (*r* = .11, *p* = .009). The spawning zone variable was dropped because single males often mated with females located in both upstream and downstream zones and not just with females located in the spawning zone they were first observed in (based on genetic identification of female mates associated with each male).

## 3 RESULTS

### 3.1 Sample collection

Over 7 years, 1024 capture events were recorded for 252 unique female and 347 unique male lake sturgeon spawners (Table 1; range 101–225 across years) in the upper Black River. Sex ratios were consistently male‐biased each year (males accounted for 61.7%–75.2% of total adults or 1.61–3.04 males per female across years; Table 1). Over 7 years, 34,969 larval lake sturgeon were captured downstream of the spawning areas (range 437–16,417 each year; Table 1) reflecting considerable interannual variation in spawning adult abundance and mortality from egg to the larval stage. On average 9.9% of sampled larvae were genotyped (a total of 3436 larval genotypes; Table 1).

**FIGURE 2:**
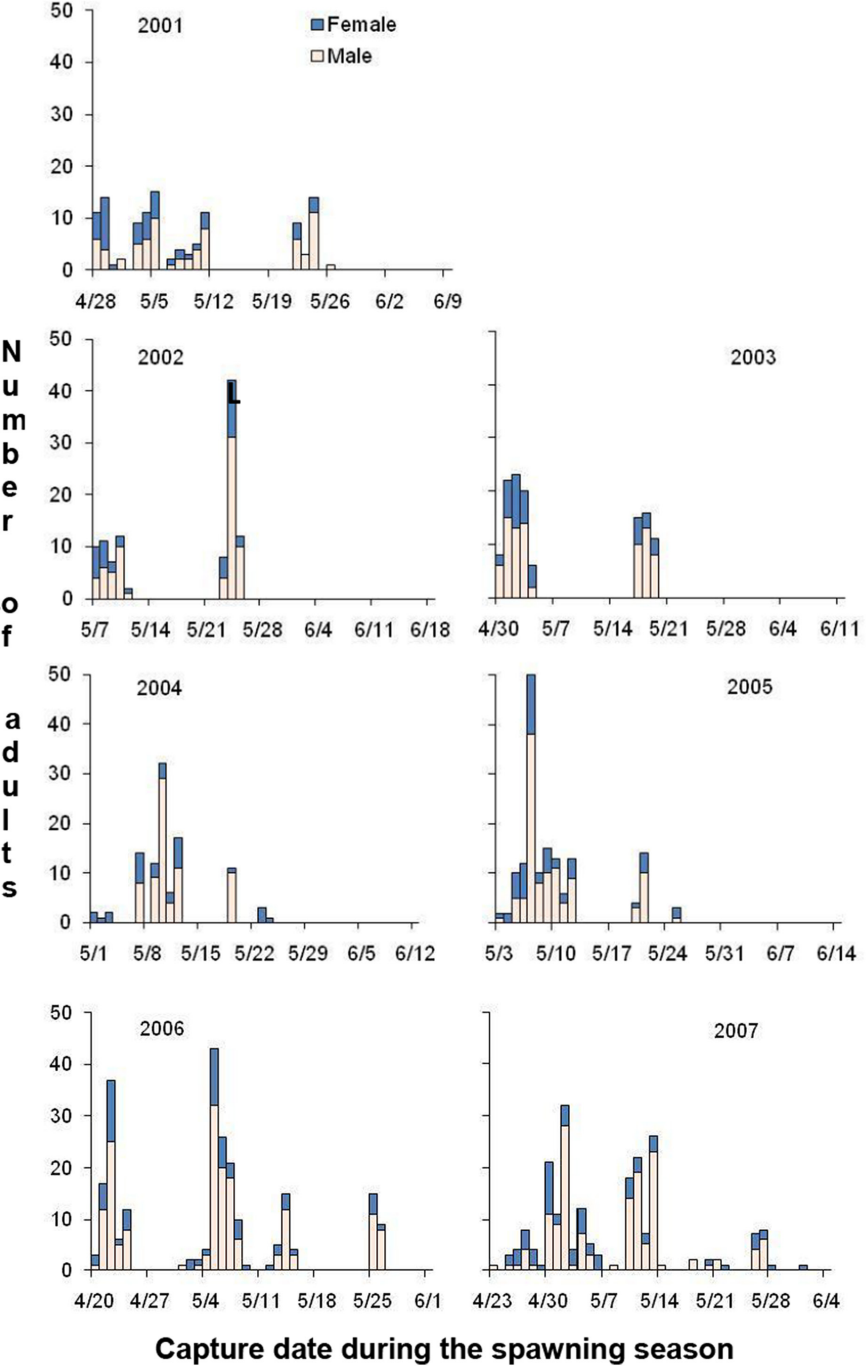
The number of adult males and females captured by day of the spawning season during 7 years (2001–2007) in the upper Black River, MI.

**FIGURE 3:**
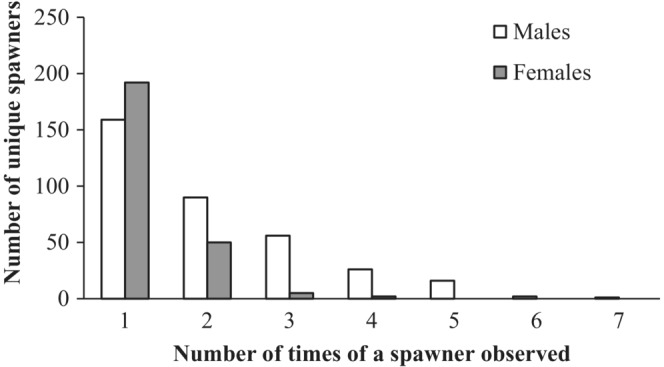
Histogram distribution of the number of unique spawners observed at different times (years) over 7 years (2001–2007).

The sampled numbers of adults spawning by year ranged from 71 to 162 males and 25 to 63 females (Table 1) which was estimated to be approximately 56.8% and 63.7% of the entire spawning group averaged across all years (Table 1). Frequency histograms (Figure 2) show interannual variation in the date of capture and presumed initiation of spawning (range April 20 to May 7), duration of spawning (range 18–43 days), and distributions of spawning numbers of males and females by date across the 7 years. Lake sturgeon spawner abundance was typically higher early in the season compared with abundance later in the season (Figure 1). The sum of adult numbers spawning over the years (1024) was substantially more than the number of unique spawners (599) because a portion of individuals (54.5% males and 22.9% females) were observed to spawn more than one time or year (range 2–7 times; Figure 3).

### 3.2 Parentage assignment

Information pertaining to the multi‐locus genotype data and parentage analyses was detailed in Duong et al. (2013). In brief, 12 microsatellite loci used for parentage analysis had moderate levels of allelic diversity (range from 2 to 11 alleles per locus), mean per locus expected heterozygosity (~0.59), and mean number of alleles per locus of ~5 (Table S1). Based on CERVUS output based on likelihoods of maternal and paternal assignment, nonexclusion probabilities (the mean probability that genetic data will fail to exclude one or a pair of unrelated candidate parents from parentage) for parental pairs over all loci was <4*10^−5^ across years (Table S1). The parental allocation correctness (the probability that each allocation was correct) from PASOS was nearly constant across 7 years, ranging between 80% and 83% and the standard deviation among iterations simulated was small (~1%). Concordance in parentage assignment between the two programs ranged from 79.2% to 85.2% of larvae assigned across 7 years. High proportions of captured males (57%–94%) and captured females (83%–98%) were concordantly assigned offspring based on the two programs. Only genotyped larvae, where one or both adults were assigned as parents concordantly in both programs, were used for analyses of reproductive success.

### 3.3 Evaluating random effects on male and female reproductive success

As a preliminary step toward identifying important fixed effects (i.e., spawning date for females) or residence time (for males), standardized body size, and reproductive zone (for females), we evaluated the random effects to include in the model based on a model with all the fixed effects. Three random effects including year, individual observation each year (Obs), and individual fish (i.e., consistent term for an adult that applied every year it was captured [Ind]) were evaluated as factors affecting the reproductive success of lake sturgeon females and males. For sex‐specific models containing all the fixed effects (Table 2), the random effects model with the lowest AIC included random effects of observation (Obs) and Year for females and all three random effects for males. For females, the model with all three random effects had nearly as low an AIC as the best model, whereas for males, the model with only random effects for Obs and Year was nearly as good as the model with all three effects. These results did not provide a compelling case for consistent (over years) among individual variation in reproductive success, beyond that which could be tied to the fixed effects in the model. For example, a fish body size remained large for multiple years, and the same individuals might tend to spawn in the same zone and at comparable times across years (Forsythe et al., 2012). However, because the random effect models with as well as without Ind as a random effect were plausible (AIC the best or close to best), we fit the alternative fixed effect models for both the competitive random effect models.

**TABLE 2:** AIC differences for models including different combinations of random effects: Year (2001–2007), individual observation each year (Obs), and individual fish (Ind) associated with reproductive success of lake sturgeon females and males.

Model	AIC difference	
	For females	For males
No random effects	1331.8	591.7
Year	722.4	228.6
Ind	197.7	310.3
Obs	54.4	130.0
Obs + Ind	54.8	132.0
Ind + Year	70.8	30.5
Obs + Year	0.0	1.1
Obs + Ind + Year	1.9	0.0

*Note*: In all models all fixed effects (standardized spawning date [for females] or standardized residence time [males], standardized body size and for females reproductive zone) were included. The lowest AIC model has an AIC difference of zero and AIC differences for other models are relative to that model.

### 3.4 Evaluation of fixed effects for female reproductive success

**FIGURE 4:**
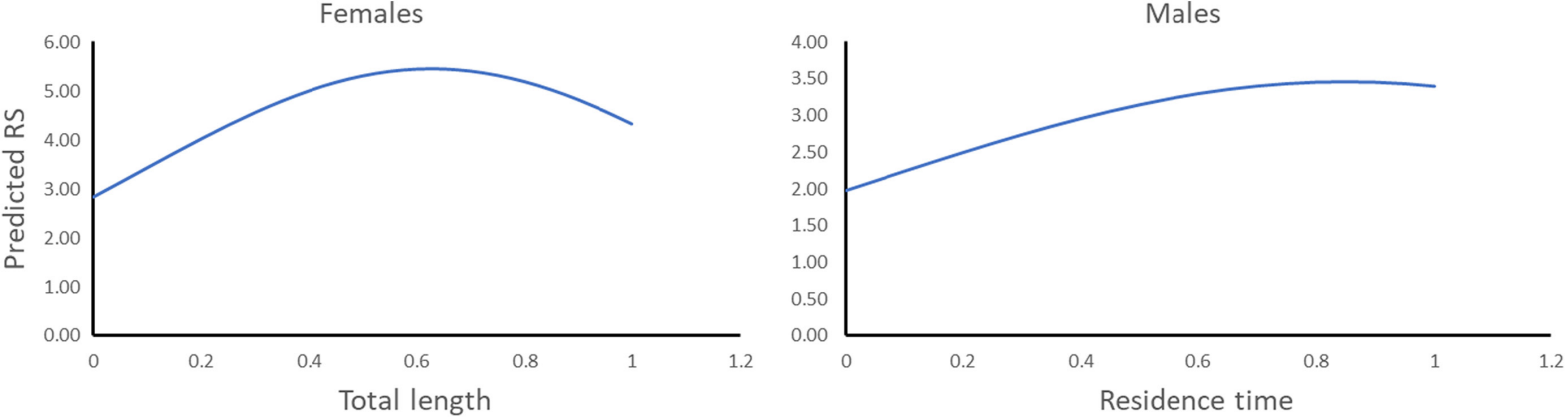
The relationships between predicted reproductive success (RS) and standardized body size for females (based on parameters in Table 4), and standardized time on spawning grounds (Residence Time) for males (based on parameters in Table 6). The relationship for females is shown for Zone 1 (no other variables were included in the lowest AIC model for males).

AIC results for fixed effects were similar for the two random effect models considered (Table 3), and estimated coefficients for fixed effects were similar (results not shown) regardless of which random effects model was considered. Consequently, we focused only on AIC results for females based on the model with Obs and Year, which was the best random effects model when all fixed effects were included (Table 2). The best model and all models within the three AIC units of the best model included reproductive zone, strongly indicating that this variable influenced female reproductive success (Table 3). The best model also included female body size. The second‐best model, with nearly as low an AIC as the best, also included female body size, and in addition included Date. The two other models within three AIC units of the best model (but just barely) included only Zone or Zone and Date. Thus, the results provided moderately strong but not overwhelming evidence for an effect of body size and weak evidence for an effect of spawning date. Parameter estimates of the best model (including random effects of Obs and Year) indicated that the predicted reproductive success of lake sturgeon females had a nonlinear relationship with body size (i.e., standardized body size) that peaked above the midpoint of observed standardized values (Table 4, Figure 4). Under this best model, female reproductive success varied among reproductive zones, with higher reproductive success for females that spawned in zones 1, 3 (upstream), and 6 (downstream). Similar patterns among reproductive zones and for the effect of standardized body size were seen for all models within three AIC of the best model that included these effects (results not shown).

**TABLE 3:** Comparison of different models for female reproductive success containing subsets of fixed effect factors, based on AIC difference.

Fixed effect factors	AIC difference	
	Obs + year RE models	Obs + Ind + year RE models
No factors (intercept only)	5.1	4.6
Date	6.8	6.7
Zone	2.8	3.0
Total length	3.7	3.1
Date + Zone	2.9	3.5
Date + Total length	5.7	5.3
Total length + Zone	0.0	0.0
Date + Total length + Zone	0.3	0.8

*Note*: The full set of fixed effect factors included standardized spawning date (Date), standardized female body size, and reproductive zone (Zone). The continuous factors (Date and Total length) included both a linear and quadratic term. AIC differences are calculated versus the lowest AIC model, which has an AIC difference of 0. AIC results for fixed effects are shown for the two lowest AIC random effect (RE) models from Table 2.

**TABLE 4:** Coefficient estimates and standard errors, predicted values, and estimated random effect variances for the model relating female reproductive success to predictors.

Parameters	Estimate	SE
Intercept	1.04	0.33
Total length	2.09	0.87
Total length squared	−1.67	0.82
Reproductive zone		
Zone 2	−0.29	0.22
Zone 3	0.15	0.30
Zone 4	−0.17	0.21
Zone 5	−0.56	0.21
Zone 6	0.04	0.29
Back‐transformed predicted values for different zones (for total length = 0.5)		
Zone 1	5.31	
Zone 2	3.96	
Zone 3	6.16	
Zone 4	4.48	
Zone 5	3.03	
Zone 6	5.55	
Back‐transformed predicted value for body size extremes and midpoint each year (for Zone 1)		
TL = 0	2.83	
TL = 0.5	5.31	
TL = 0.1	4.33	
Estimated variances for random effects		
Observation	0.66	
Year	0.25	

*Note*: Results are for the lowest AIC fixed effect model (Table 3) among models using the lowest AIC random effect model (Table 2). The best model included standardized body size (and its square) and reproductive zone as predictors and included Observation and Year as random effects.

### 3.5 Evaluation of fixed effects for male reproductive success

As for females, two random effect models, one with all three random effects and one that excluded the individual effect, were plausible (within three AIC of the best model), but in the case of males, the lower AIC model had all three random effects. We focused on the evaluation of fixed effects for the random effects model that included all three variables, although model choice among fixed effects did not depend on the random effects models, and as for females estimated fixed effect parameters were similar regardless of the random effects model (results not shown).

**FIGURE 5:**
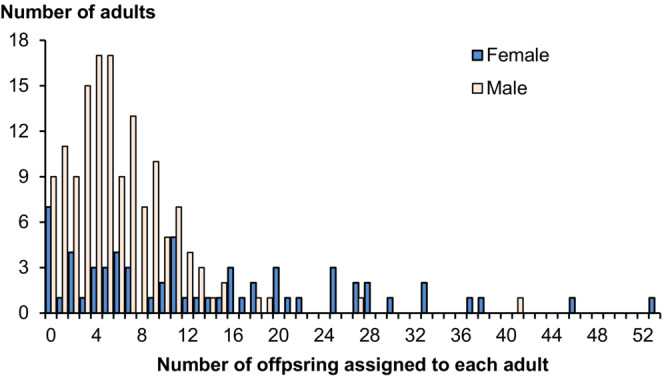
Distribution of the number of offspring assigned to each individual male and female lake sturgeon captured in 2007.

Reproductive success for males and females was skewed (Figure 5). For males, there was clear evidence that residence time in the spawning areas influenced reproductive success (Table 5). Based on the best model, male reproductive success increased with increasing residence time in the spawning areas past the midpoint residence time, then declined somewhat but still was about 70% higher for males with the longest residence time compared to males with the shortest residence time (Table 6, Figure 4). The second‐best and only other competitive model (within three AIC of best) estimated similar patterns for the effects of residence time (results not shown) but also included male body size (Table 5).

**TABLE 5:** Different models showing effects of fixed factors on male reproductive success.

Fixed effect factors	AIC differences	
	Obs + year RE models	Obs + Ind + year RE models
No fixed effects	22.9	23.7
Total length	23.7	25.0
Residence time	0.0	0.0
Residence time + Total length	1.1	1.9

*Note*: Comparison of different models for male reproductive success containing subsets of fixed effect factors, based on AIC differences. The fixed effect factors were standardized male body size and standardized residence time (Residence Time). These continuous factors included both a linear and quadratic term. AIC differences are calculated versus the lowest AIC model, which has an AIC difference of 0. AIC difference results are shown for the two best random effect models (Table 2).

**TABLE 6:** Coefficient estimates and standard errors, predicted values, and estimated variances for random effects for the model relating male reproductive success to predictors.

Fixed effect coefficients	Estimate	SE
Intercept	0.68	0.18
Residence time	1.31	0.45
Residence time squared	−0.77	0.46
Back‐transformed predicted values for extremes and midpoint of standardized residence time		
Residence time = 0	1.98	
Residence time = 0.5	3.15	
Residence time = 1	3.40	
Estimated variances for random effects		
Observation	0.16	
Individual	0.07	
Year	0.18	

*Note*: Shown are results for the lowest AIC fixed effect model (Table 5) among models using the lowest AIC random effect model (Table 2). The best model included standardized residence time on spawning grounds (and its square) as a predictor and included Observation, Individual, and Year as random effects.

## 4 DISCUSSION

We quantified the variation in, and factors affecting male and female reproductive success for lake sturgeon, a long‐lived iteroparous fish of conservation concern over a period of 7 years constituting multiple reproductive events for much of the adult population (Forsythe et al., 2012). For each sex, the most reproductively successful individuals in 1 year were not consistently more successful than other individuals in other years once fixed explanatory factors were accounted for (i.e., lack of random Ind effect; Table 2). Repeatability of comparatively higher or lower reproductive success for individual males and females appears to be attributed to the fixed effects associated with where individuals spawned (i.e., reproductive zone) when individuals spawned (i.e., standardized spawning date), standardized body size, and for males related to river residence time which affects intersexual encounter levels (Larson et al., 2020) which were repeatable across years (Forsythe et al., 2012). Data indicate that the population was not composed of reproductively isolated groups as previously inferred based on direct observations of high individual repeatability of spawning date and location for males and females (Forsythe et al., 2012) due largely to extended male residence times spanning multiple spawning runs. However, findings of a high occurrence of matings between adults of seemingly temporally reproductively isolated “early” and “late” spawning groups (i.e., reproductive isolation by time; Hendry & Day, 2005) has considerable implications for the relative reproductive success of adults spawning at different times under future climatic‐induced changes (e.g., diel mean temperature differences can exceed 10°C over the spawning period; data not shown), for population effective size, and retention of population levels of genetic diversity given estimated heritabilities of offspring size and growth (Dammerman et al., 2015, 2016). The potential for greater future interindividual adult variance in reproductive success is likely for sturgeons as they are threatened or endangered globally, with many populations in low abundance or continually declining (Congiu et al., 2023), and affects reproductive success may be accentuated, in part due to depensatory effects (Dammerman et al., 2019).

### 4.1 Factors influencing male and female reproductive success

Males that spent long periods on the spawning ground had higher reproductive success than males spawning with females from the same spawning group (early males with early females and late males with late females). Context‐dependent male modification of behavior is commonly observed in a taxonomically diverse array of species, including fishes generally (DeWoody & Avise, 2001; Forsgren et al., 2002; Martin & Taborsky, 1997), and Atlantic salmon (*Salmo salar*) specifically (Landry et al., 2001), fruit flies (D*rosophila melanogaster*, Bateman, 1948), and sea urchin (*Strongylocentrotus franciscanus*; Levitan, 2004). In this study, on average, ~27% of adult males produced offspring that were genotyped from mates of different (early vs late) spawning groups.

Our findings suggest several questions that deserve additional study. First, if greater male residence time allows greater access to mates and greater reproductive success, why do not a higher proportion of males engage in this behavior? Second, given we documented high interannual variation in the percentage of males that engage in between‐group spawning (range 10.2%–49.7%), what biological or physical stream conditions are associated with this behavioral plasticity? Based on visual inspection of data on the numbers of adults spawning each day of the season (Figure 2), the length of the spawning season (range 18–43 days) and day of first spawning (range April 20 to May 7) were not related to interannual variation in male residence time or proportions of intergroup mating. Calculations of mean residence time and proportion of intergroup matings could be evaluated using simple correlation. However, the number of years is too low to warrant doing this formally.

Clearly, some costs of reproduction seem to have been incurred to deter a greater number of males from consistently having long residence times. Allocation of energy to reproduction can incur significant costs to future reproduction (Reznick, 1985). One measure of the reproductive cost we were able to evaluate recently was interspawning intervals which varied significantly as a function of male spawning behavior (Larson et al., 2020). Males that remained at the spawning site longer and successfully mated with females from both early and late spawning dates were more likely to skip spawning the following year compared with males that successfully spawned with only females from a single spawning group (data not shown). Given that interbreeding intervals and the number of reproductive bouts in a lifetime dictate lifetime reproductive success in long‐lived iteroparous species (Kruuk et al., 1999; Pianka & Parker, 1975), costs and benefits of greater or less reproductive effort in the current year (i.e., reflected in male residence time) appear to trade‐off with future reproductive potential.

### 4.2 Challenges to describing reproductive success for long‐lived iteroparous species

Spawning synchrony and mate availability in terms of abundance and operational sex ratios (Table 1) are important factors contributing to levels of intersexual encounters and to gamete fertilization success, and thus reproductive success in lake sturgeon (Dammerman et al., 2019) as well as other broadcast‐spawning species (Arnold & Duvall, 1994; Emlen & Oring, 1977; Levitan & Petersen, 1995). Therefore, spawning date and spawning location should be, and was, related to reproductive success for females. Because lake sturgeon spawning early in the season were usually more numerous and residence time of males that spawned early was over longer periods compared with individuals spawning later in the season (Figure 1), one might expect that reproductive success would be higher for individuals spawning early in the season and at upstream locations compared with individuals spawning late in the season and downstream locations. Further, more larvae were produced early in the dispersal period (data not shown), suggesting at first consideration that reproductive success would be highest for females spawning upstream early in the spawning season. However, our results do not entirely support this expectation. Results highlight the important distinction between reproductive output at the population or subpopulation level and per‐capita reproductive success. There are more spawning adults and thus larger overall numbers of larvae produced early in the season, though per‐capita adult reproductive success was greater in later spawning adults.

Environmental factors, while not explicitly evaluated, are associated with spawning date and spawning locations used by adults and can affect the probability of survival of fertilized eggs to larval stages and thus could contribute to variation in reproductive success at different spawning locations and during a spawning season. Factors associated with early life stage mortality (i.e., before larvae are captured during dispersal) include biotic factors, such as predation (Waraniak et al., 2019), microbial infection (Fujimoto et al., 2020), food availability, etc., and abiotic factors (Dammerman et al., 2019), for example, water temperature and discharge (direct or indirect effects associated with oxygen supply; Caroffino et al., 2010; Kamler, 2005). Temperature and discharge in the Upper Black River, where lake sturgeon spawn, follow a seasonal pattern in which lower temperature and higher discharge characterize the stream early in the spawning season compared with later in the season (Forsythe et al., 2012). As variation in reproductive success will be driven by extrinsic factors (physical environmental variables) affecting all individuals, like stream temperature and discharge, future studies could profitably estimate the relative contribution of these factors to observed variation in reproductive success.

Adult body size is another attribute that was predicted to contribute to variation in lake sturgeon reproductive success. Similar to other fish species, body size, and fecundity of lake sturgeon females are positively related (Bruch et al., 2006). However, the high reproductive potential may not alone predict high reproductive success because early life mortality is related to important environmental factors associated with the timing and location of spawning, particularly discharge and temperature (Dammerman et al., 2020). We found that the reproductive success of female lake sturgeon increased with increasing body size, at least until a female well exceeded the median body size for fish reproducing in a year. The trend in data was not large, however, despite estimates that female fecundity scales linearly with body size (Bruch et al., 2016). The increase in male reproductive success with body size was modest and could be explained by several hypotheses. First, larger males might have higher sperm quantity or/and quality (e.g., guppy *Poecilia reticulata* (Skinner & Watt, 2007), lake whitefish *Coregonus clupeaformis* (Blukacz et al., 2010)). Alternatively, larger males might compete better for mates, leading to higher reproductive success (e.g., in Atlantic cod, Rowe et al., 2007; leopard grouper *Mycteroperca rosacea*, Erisman et al., 2007). Last, because females release thousands of eggs into fast‐flowing water (Finley et al., 2019) that are widely dispersed over stream substrates (Dammerman et al., 2020), female behavior (e.g., cues that elicit male aggregation immediately prior to egg release) may allow large numbers of attending males to release sperm coincident with oviposition.

We did not document evidence to distinguish between the aforementioned variables or other possibilities. For lake sturgeon and oviparous fishes generally, larger males are better able to position themselves in proximity to females during spawning and thus may increase reproductive success (Bekkevold et al., 2002; Erisman et al., 2007; Petersen & Warner, 1998). In leopard groupers (*Mycteroperca rosacea*), for example, male–male competition occurs where dominant males who occupy the closest position to females can fertilize more eggs and, therefore, realize higher reproductive success than peripheral males (Erisman et al., 2007). Male‐biased sex ratios in spawning groups observed in lake sturgeon (Figure 2) suggest an opportunity for male–male competition (Hall & Hanlon, 2002; Sadovy et al., 1994). The positive fixed effect of male body size could be attributed to intrasexual competition (Andersson, 1994; Roff, 2002), where high‐quality males choose (or have access to) high‐quality females, but lower‐quality males must “make do” with lower‐quality females (Baldauf et al., 2009; Kokko & Mappes, 2005).

Mating among individuals might not be random with respect to arrival time, especially for broadcast‐spawning species where spawning synchrony is an important determinant of reproductive success (Emlen & Oring, 1977; Levitan & Petersen, 1995). We found the majority (~70%) of mating pairs where males and females were captured <8 days apart. Because egg expulsion typically lasts for only 8–12 h (Bruch & Binkowski, 2002) or a few days (Forsythe et al., 2012), mating between members from different groups is likely driven by male behavior, especially residence time on spawning areas, unless females experience stressful conditions such as high discharge or cold water (Dammerman et al., 2019). We observed, for example, that 14.7% of males in 2007 were recaptured (the second time) after a period of 7–16 days following the first capture. Many recaptured males had left the spawning areas entirely and returned with a new group of females. The behavior of returning or retaining for longer periods at the spawning areas potentially increases opportunities to mate with females arriving later in the season (Larson et al., 2020). Therefore, high male stream residence time dilutes reproductive isolation over time. In other lake sturgeon populations, the same connection between male behavior of prolonged occupancy of spawning areas and mating opportunities with multiple females was also observed (Bruch & Binkowski, 2002). Our measure of reproductive success was based on the number of recovered larvae downstream from the spawning grounds that were assigned to parents. Some collected larvae were not assigned to parents, likely because not all parents were physically handled. Thus, our focus was on reproductive success given a parent was handled during sampling. In addition, it is likely that not all parents that produced offspring were reflected in the genotyped larval samples because not all larvae were sampled and not all sampled larvae were genotyped in all years. Results point to how variation in explanatory factors within a year influences the relative reproductive success of different individuals that year and we addressed this by standardizing our measures of adult size and spawning location and including a random year effect in the analysis 0.75% accurate. In corroboration with the simulations conducted by Harrison et al. (2013), the high concordance of assignments across two independent statistical approaches to parentage assignment were evidence that associated estimates of reproductive success were accurate (e.g., Sard et al., 2016; Walling et al., 2010). The above evidence suggests that although there were likely some errors in parent‐offspring assignment, there was no evidence that errors occurred in a systematic manner that strongly biased estimates of reproductive success in a manner that would affect which predictors were significant in regression analyses. Thus, most assignments were likely correct, and associated estimates of reproductive success are likely strongly correlated with individual fitness.

### 4.3 Implications of results for species management

Components of the mating system such as inter‐ and intrasexual behavior play an important role in population recruitment and abundance (Rowe & Hutchings, 2003). The benefits of longer retention times by males accrue as a function of the increase in the number of female interactions (Larson et al., 2020) and the increase in numbers of offspring sired (this paper). However, there can be costs as well. The “costs” include a reduction in sperm quality (Larson et al., 2020) and a longer interspawning interval (Larson, 2023) that can potentially decrease life‐time reproductive success. Behaviors also can influence levels of population genetic diversity and levels of genetic structure (Fagan et al., 2010; Johannesen & Lubin, 1999).

Life history theory predicts (Pianka & Parker, 1975) when considering concepts of reproductive value (Fisher, 1930) which is age‐specific expectations of all present and future offspring, that there would be an inverse relationship between levels of investment in current reproduction (of which “river retention time” in our analysis is a part), and the likelihood of future reproduction (residual reproductive success). Thus, because “younger” males have higher expectations of future reproductive success (more future spawning events), they should invest less in current reproduction than older males.

Results from our long‐term capture–recapture efforts and genetic determination of parentage from our wadable stream system warrant comparisons to other studies from other sturgeon species and other physical environmental context. Work published for lake sturgeon, largely based on telemetry data in other (Izzo et al., 2021; Thiem et al., 2013) and larger riverine environments (Kessel et al., 2018) indicate that variation in spawning behavior during the reproductive season is common. Likewise, intrapopulation diversity in migratory and spawning behavior has been documented in larger river systems and in different sturgeon species, for example, white sturgeon (*Acipenser transmontanus*) in the Kootenay River (Paragamian & Kruse, 2001).

Results from this study increase our understanding of variation in, and factors contributing to reproductive success in the polygamous mating system of a long‐lived iteroparous species of conservation concern. Estimation of the proportion of adults contributing offspring to the larval stage and quantifying reproductive success provides parameters necessary for the estimation of the effective number of breeding adults (*N*_b_, Duong et al., 2013) and helps explain interannual variability in estimates of *N*_b_; both of which are important population parameters used for conservation and management.

Two important implications can be drawn from findings quantifying aspects of lake sturgeon reproductive behavior. First, reproductive success among individuals was highly skewed (Figure 5), which likely resulted in a lower effective population size compared with the population census size. Effects of male and female behavior in the current year can be countered by longevity and iteroparity with respect to maintaining higher effective population size, and ultimately retaining the genetic diversity of the population (Lippé et al., 2006). Second, data in this study showing that on average 30% of males may mate with females in both early and late spawning groups indicate that male‐mediated gene flow between temporally semi‐isolated and habitually ‘early’ and ‘late’ spawning females is a homogenizing influence. Matings between individuals in different spawning groups serve to maintain gene flow among groups that routinely spawn at different times (Forsythe et al., 2012), thereby decreasing the potential for genetic differentiation among groups within this population. This result also contributes to the effective management of the population as understanding reproductive behavior across the entire spawning season and its outcomes on population dynamics is critical for conservation efforts (e.g., protection from poaching or maintaining baseline flows through river regulation). Information pertaining to the degree of plasticity in male behavior with respect to the duration of time spent in and near spawning areas is important, as behavioral plasticity and the ability of individuals to modify times and locations used for reproduction may become increasingly challenged due to climate‐induced variability in environmental conditions.

## AUTHOR CONTRIBUTIONS

**Thuy‐Yen Duong:** Conceptualization (equal); data curation (lead); formal analysis (supporting); methodology (supporting); validation (supporting); writing – original draft (equal); writing – review and editing (equal). **James Bence:** Conceptualization (supporting); formal analysis (lead); investigation (supporting); methodology (supporting); validation (supporting); writing – original draft (supporting); writing – review and editing (supporting). **Patrick S. Forsythe:** Conceptualization (supporting); investigation (supporting); writing – review and editing (equal). **James A. Crossman:** Conceptualization (supporting); data curation (supporting); formal analysis (supporting); investigation (supporting); methodology (supporting); validation (supporting); writing – review and editing (equal). **Edward A. Baker:** Funding acquisition (supporting); investigation (supporting); project administration (supporting); resources (supporting); writing – review and editing (supporting). **Nicholas M. Sard:** Formal analysis (equal); methodology (supporting); software (supporting); visualization (supporting); writing – review and editing (supporting). **Kim T. Scribner:** Conceptualization (equal); formal analysis (supporting); funding acquisition (lead); investigation (lead); methodology (supporting); project administration (lead); resources (lead); supervision (lead); writing – original draft (supporting); writing – review and editing (lead).

## FUNDING INFORMATION

Funding for the project was provided by the Michigan Department of Natural Resources (DNR), the Great Lakes Fisheries Trust, Michigan State University Ag Bio Research, and the Partnership for Ecosystem Research and Management (PERM) cooperative program between the Michigan DNR and the Department of Fisheries and Wildlife at Michigan State University. TYD was supported by a Vietnamese Government Fellowship (Project 322).

## CONFLICT OF INTEREST STATEMENT

The authors declare that they have no conflict of interest to disclose.

## Supporting information


Table S1.

Figure S1

## Data Availability

Data associated with the study are currently deposited in DRYAD entry https://doi.org/10.5061/dryad.72242.
