# Supplementary material for: Individual‐based analyses reveal effects of behavioral and demographic variables associated with multi‐annual reproductive success of male and female lake sturgeon
Source: Ecol Evol. 2023 Jul 12;13(7):e10253. doi: 10.1002/ece3.10253 (PMC10338754; doi:10.1002/ece3.10253)

Supplemental Figure S1. Photograph of adult lake sturgeon (*Acipenser fulvescens*) in the Black River, Michigan spawning area following capture (photo credit – Douglas Larson)


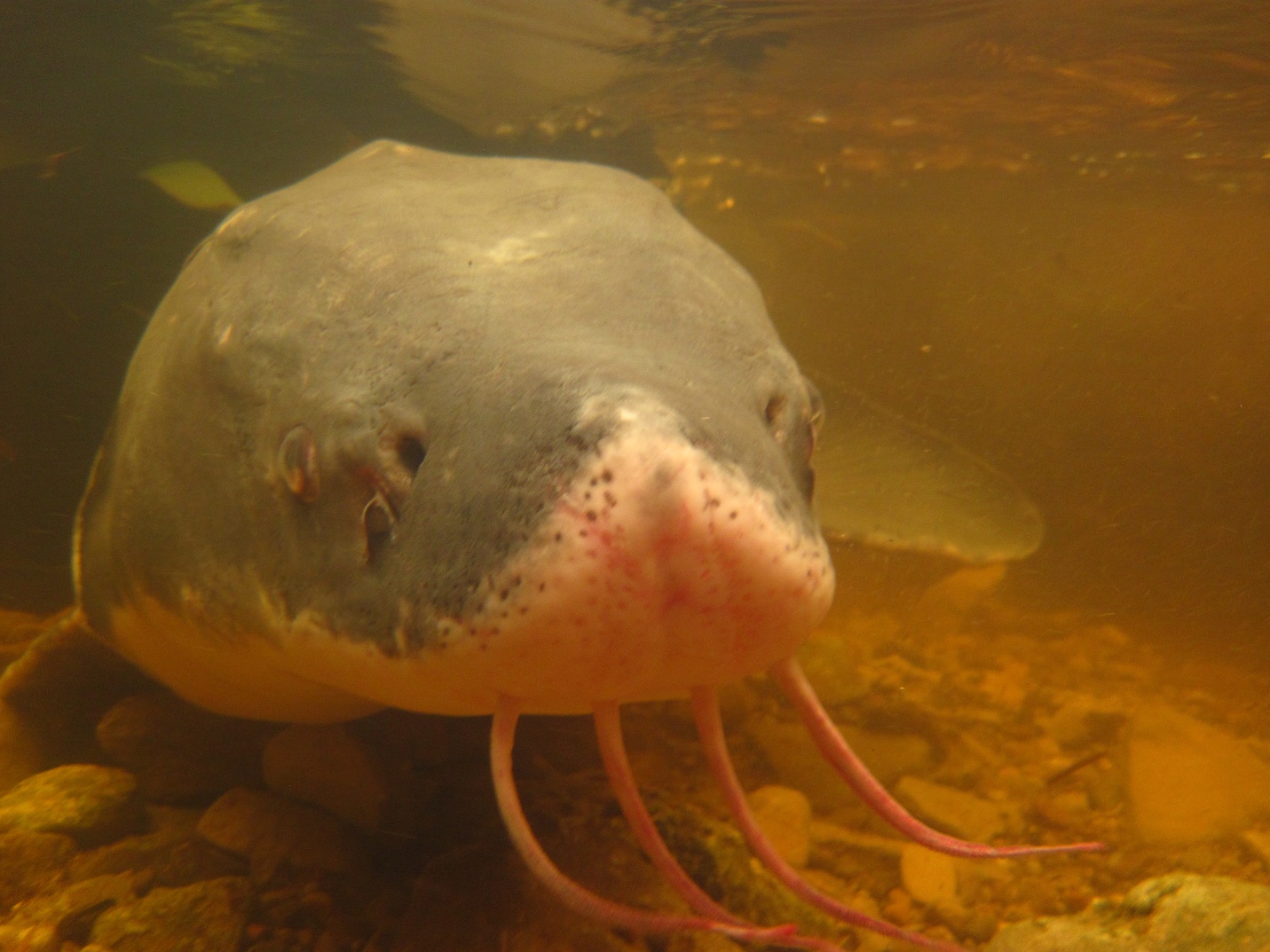

Supplement: Supplementary file 2 — Figure S1 [file ECE3-13-e10253-s001.docx]
